# Supplementary material for: Molecular Profile of Variants Potentially Associated with Severe Forms of COVID-19 in Amazonian Indigenous Populations
Source: Viruses. 2024 Feb 26;16(3):359. doi: 10.3390/v16030359 (PMC10974871; doi:10.3390/v16030359)
Supplement: Supplementary file 1 [file viruses-16-00359-s001.zip › viruses-2790204-Supplementary Materials.pdf]

Supplementary Table S1. Description of the 17 variants with low impact predicted present in the genes *AQP3, ARHGAP27, ELF5, IFNAR2, LIMD1, OAS1* and *UPK1A*.

Gene	Position	SNP ID	Ref[a]	Var[b]	Impact Predicted by SNPeff	Variant Allele Frequence					
						INDG	AFR	AMR	EAS	EUR	SAS
AQP3	33442954	rs2228332	G	A	LOW	0,945313	0.7462	0.7106	0.7177	0.5901	0.5756
AQP3	33447426	rs591810	C	G	LOW	0,96875	0.7678	0.8105	0.7486	0.703	0.6534
AQP3	33442882	*	G	A	LOW	0,083333	-	-	-	-	-
AQP3	33443875	rs114247802	G	A	LOW	0,083333	0.0048	0.0007	-	-	-
ARHGAP27	45396708	rs1297259327	C	T	LOW	0,083333	-	-	-	-	-
ARHGAP27	45430037	rs7220206	G	A	LOW	0,028571	0.2174	0.4237	-	0.4716	0.4716
ELF5	34480798	rs2231828	T	C	LOW	0,046875	0.0806	0.1829	0.027	0.4214	0.3137
IFNAR2	33262774	rs34865572<br>rs750263757	CT	C	LOW	0,137931	1	1	1	1	1
IFNAR2	33244934	rs149186597<br>rs79402470	TTT<br>C	T	LOW	0,083333	0.0026	0.0116	0.0003	0.0254	0.0261
IFNAR2	33263275	rs147568312	C	T	LOW	0,083333	0.0163	0.0004	0.0002	-	-
LIMD1	45636143	rs11379880<br>rs5848761	C	CT	LOW	1	1	1	1	1	1
LIMD1	45595761	rs267237	C	T	LOW	0.624	0.5847	0.7219	0.9384	0.5625	0.6755
LIMD1	45595947	rs267236	T	C	LOW	0,945313	0.7101	0.7409	0.9385	0.6046	0.6991
LIMD1	45636143	rs11379880<br>rs5848761	C	CT	LOW	1	1	1	1	1	1
LIMD1	45636146	rs5848762<br>rs869033908	CA	C	LOW	1	1	1	1	1	1
OAS1	1,13E+08	rs7955146	C	T	LOW	0,013514	0.3015	0.0159	-	0.0015	0.0005
UPK1A	35677970	rs2285420	G	A	LOW	0,166667	0.0694	0.1678	0.1273	0.1397	0.1087

[a]Reference Allele; [b]Variant Allele; *Variants without described SNP; (-) – No annotation; INDG: Indigenous population; AFR: African population; AMR: American population; EAS: East Asian population; EUR: European population; SAS: South Asian population.

Supplementary Table S2. Comparison of the allele frequency of the INDG population with the continental population (AFR, AMR, EUR, EAS and SAS).

Gene	SNP ID	INDG vs. AFR *	INDG vs. AMR *	INDG vs. EAS *	INDG vs. EUR *	INDG vs. SAS *
AQP3	rs2228332	0.00018	3.693×10^{-5}	5.348×10^{-5}	4.293×10^{-9}	9.082×10^{-10}
AQP3	rs591810	3.963×10^{-5}	0.00075	1.031×10^{-5}	3.214×10^{-7}	1.087×10^{-8}
AQP3	rs2231235	0.09707	0.00324	0.42487	5.369×10^{-11}	1.929×10^{-6}
AQP3	rs114247802	0.00019	7.994×10^{-5}	NA	NA	NA
AQP3	rs2231231	0.00117	0.00010	5.442×10^{-5}	1.620×10^{-7}	2.264×10^{-9}
AQP3	rs12555686	0.63286	6.573×10^{-8}	2.387×10^{-5}	0.00362	0.00356
AQP3	rs16919255	0.26429	0.00027	1.570×10^{-5}	0.14649	0.14583
ELF5	rs2231825	0.09707	0.00324	0.42487	5.369×10^{-11}	1.929×10^{-6}
ELF5	rs2231821	1.000	0.01790	0.42487	6.224×10^{-7}	3.250×10^{-5}
ELF5	rs2231828	0.46491	0.00498	0.42487	1.168×10^{-10}	1.051×10^{-6}
ELF5	rs737254	4.787×10^{-6}	6.137×10^{-7}	1.000	1.465×10^{-12}	1.611×10^{-7}
UPK1A	rs747589460	0.65377	1.000	1.000	0.56369	0.20915
UPK1A	rs75289222	0.41762	0.04259	0.07032	0.07073	0.21094
UPK1A	rs2267586	0.00344	0.14912	0.63094	0.05448	0.05570
UPK1A	rs2285421	3.016×10^{-16}	3.180×10^{-5}	1.341×10^{-6}	0.00030	1.562×10^{-11}
UPK1A	rs2285420	0.01134	1.000	0.32717	0.45227	0.14569
OAS1	rs7968145	1.000	0.14897	1.000	0.01497	0.09495
OAS1	rs1131476	0.02583	4.458×10^{-5}	7.665×10^{-7}	3.188×10^{-11}	6.497×10^{-10}
OAS1	rs1131454	3.523×10^{-11}	0.37592	0.78508	0.34783	0.34646
OAS1	rs2660	0.02583	7.618×10^{-5}	1.374×10^{-6}	1.525×10^{-10}	2.482×10^{-9}
OAS1	rs10774671	3.039×10^{-10}	0.73237	0.51581	0.00451	0.03860
OAS1	rs7967461	0.01055	3.650×10^{-8}	1.260×10^{-7}	1.118×10^{-14}	6.043×10^{-10}
OAS1	rs11352835	0.01087	3.432×10^{-8}	2.366×10^{-7}	3.623×10^{-14}	6.043×10^{-10}
OAS1	rs7955146	1.422×10^{-8}	1.000	NA	0.21318	0.11573
OAS1	rs1051042	0.02583	4.458×10^{-5}	7.665×10^{-7}	3.220×10^{-11}	5.950×10^{-10}
LIMD1	rs267237	1.489×10^{-9}	6.326×10^{-5}	1.000	3.942×10^{-10}	3.273×10^{-6}
LIMD1	rs267236	2.363×10^{-5}	0.00028	1.000	9.931×10^{-9}	1.806×10^{-5}
ARHGAP27	rs7222444	0.02289	0.00064	0.11267	1.540×10^{-6}	0.10633
ARHGAP27	rs2959953	6.052×10^{-7}	4.116×10^{-5}	0.03356	1.238×10^{-7}	6.31360×10^{-6}
ARHGAP27	rs62064597	0.19781	0.30226	1.57513×10^{-5}	0.00765	0.80278
ARHGAP27	rs7220206	0.00011	2.062×10^{-11}	NA	$7.884 X 10^{-14}$	$2.465 X 10^{-11}$
ARHGAP27	rs115993362	0.13752	0.00129	NA	1.589×10^{-5}	1.801×10^{-5}
ARHGAP27	rs35327136	0.00401	0.00022	NA	795.025	0.16105
ARHGAP27	rs142163608	0.00890	NA	NA	NA	$1.801 X 10^{-5}$
ARHGAP27	rs184103721	0.00063	1.000	0.05055	0.00137	0.00148
ARHGAP27	rs201721078	5.129×10^{-15}	1.542×10^{-5}	NA	$1.381 X 10^{-14}$	NA
ARHGAP27	rs117139057	0.36469	0.16845	0.00951	0.37949	0.83868
IFNAR2	rs1051393	1.824×10^{-21}	1.053×10^{-5}	0.00258	1.215×10^{-11}	3.942×10^{-6}
IFNAR2	rs2229207	2.456×10^{-8}	0.00186	0.003	1.035×10^{-7}	6.205×10^{-5}
IFNAR2	rs1131668	1.229×10^{-10}	2.771×10^{-7}	0.00098	7.147×10^{-11}	9.955 x 10-7

IFNAR2	rs9984273	3.001×10^{-14}	0.00013	0.00938	3.134×10^{-10}	2.268×10^{-7}
IFNAR2	rs2834158	5.32×10^{-15}	0.00664	0.22382	3.818×10^{-7}	0.00342
IFNAR2	rs17860118	0.00771	0.15350	0.34659	0.02174	0.16154
IFNAR2	rs149186597<br>rs79402470	8.004×10^{-5}	0.00602	1.575×10^{-5}	0.04188	0.04602
IFNAR2	rs3216172<br>rs397789038	0.00380	2.699×10^{-5}	4.368×10^{-10}	0.02185	3.997×10^{-6}
IFNAR2	rs147568312	0.00890	7.994×10^{-5}	1.575×10^{-5}	NA	NA
IFNAR2	rs56197608	NA	1.000	0.62213	1.000	1.000

INDG: Indigenous population; AFR: African population; AMR: American population; EAS: East Asian population; EUR: European population; SAS: South Asian population; *p-value defined by Fisher`s exact test. Bold characters indicate a significant difference (*p-value** < 0.05).
